# Serum Immune-Inflammation Index Assessment in the Patients with Ulcerative Colitis

**DOI:** 10.1155/2022/9987214

**Published:** 2022-01-31

**Authors:** Zehra Betul Pakoz, Muge Ustaoglu, Sezgin Vatansever, Elif Saritas Yuksel, Firdevs Topal

**Affiliations:** ^1^Katip Celebi University School of Medicine, Department of Gastroenterology, Izmir, Turkey; ^2^Ondokuz Mayıs University School of Medicine, Department of Gastroenterology, Izmir, Turkey; ^3^Ataturk Training and Research Hospital, Department of Gastroenterology, Izmir, Turkey

## Abstract

Radiologic and endoscopic diagnostic methods are used to determine disease activity in ulcerative colitis (UC). In order for endoscopic procedures to be invasive and to prevent radiation exposure, especially in young people, studies have been carried out frequently to determine a simple, fast, and reliable activity marker with laboratory methods. Our aim in this study is to determine the usefulness of serum immune-inflammatory index as a noninvasive marker of activation in patients with ulcerative colitis. A total of 82 consecutive patients treated with a diagnosis of ulcerative colitis were included in the study. The disease activation was assessed using the Mayo endoscopic subscore. The site of involvement was grouped into two as left colitis and extensive colitis. Patients were divided into two groups as those who had active disease based on clinical and endoscopic findings and those who were in remission. C-reactive protein (CRP) levels, platelets, neutrophils, and lymphocytes were recorded in all participants. The systemic immune-inflammation index (SII) and CRP values were compared between UC patients with active disease or remission. The correlations between CRP, SII, and Mayo endoscopic subscores were analyzed. In addition, ROC curve analysis for SII was performed to determine the cut-off value, sensitivity, and specificity in determining ulcerative colitis activity. The value of SII was significantly higher in the active group than the remission group (respectively, 1497 ± 1300 and 495 ± 224, *p* < 0.001). In the correlation analysis, a significant correlation was found between SII and Mayo subscore. In ROC curve analysis, SII was found to be significantly effective in determining activity in ulcerative colitis patients. For 0.860 area under the curve, the sensitivity was 68.1% and the specificity was 91.2% at a cut-off value of 781.5. SII is significantly higher in patients with active ulcerative colitis than those in remission. It shows promise for use as a noninvasive marker of active ulcerative colitis.

## 1. Introduction

Ulcerative colitis (UC) is a chronic inflammatory bowel disease that can involve all colon segments starting from the rectum, causing diffuse and continuous inflammation. It progresses clinically with activation and remissions [1]. The diagnosis of ulcerative colitis is made by clinical, endoscopic findings, and histological evaluation. Determination of disease activity is important in determining the treatment of the patient [2].

Radiologic and endoscopic diagnostic methods are used to determine disease activity. In order for endoscopic methods to be invasive and to prevent radiation exposure, especially in young people, studies have been carried out frequently to determine a simple, fast, and reliable activity marker with laboratory methods [3, 4]. C-reactive protein (CRP) and fecal calprotectin as laboratory methods are currently used for this purpose [5]. Apart from UC, CRP can be elevated in many acute conditions, especially infections. Therefore, its usefulness in ulcerative colitis is limited compared to fecal calprotectin [6]. Fecal calprotectin levels are affected by bowel movements, and different results can be obtained in the following days [7]. For these reasons, the search for a reliable, fast, and easy noninvasive method to determine the activity in ulcerative colitis still continues.

The systemic immune-inflammation index (SII) is an indicator calculated using neutrophil, platelet, and lymphocyte values. It is obtained by multiplying the neutrophil count and platelet count and dividing by the lymphocyte count. A high index of this index indicates the presence of relatively high neutrophil and platelet counts and low lymphocyte counts. This is indicative of a strong inflammatory response [8]. Its relation with disease prognosis and activity in many cancer types such as pancreatic cancer, colorectal cancer, bladder cancer, and inflammatory diseases has been shown in many studies [9–13].

Our aim in this study is to determine the usefulness of serum immune-inflammatory index as a noninvasive marker of activation in patients with UC and to determine the cut-off value as an activity indicator.

## 2. Materials and Methods

### 2.1. Study Population

A total of 82 consecutive patients aged over 18 years treated with a diagnosis of ulcerative colitis in our department between January 2020 and June 2020 were included in the study. Ulcerative colitis diagnosis was based on clinical, endoscopic, and histopathology findings.

### 2.2. Study Design

The study was planned as a retrospective study. Patients' age, gender, disease duration, treatments, colonoscopy results, and laboratory results were recorded for all patients from hospital software system.

### 2.3. Colonoscopic Evaluation

The disease activation was assessed using the Mayo endoscopic subscore, 0 points for normal or inactive lesions, 1 point for mild (redness, the reduced texture of blood vessel, and mildly brittle mucosa), 2 points for moderate (significant erythema, disappeared texture of blood vessel, brittle, or eroded mucosa), and 3 points (spontaneous mucosal bleeding or ulceration) [14]. The site of involvement was grouped into two as left colitis and extensive colitis. Patients were divided into two groups as those who had active disease based on clinical and endoscopic findings and those who were in remission.

### 2.4. Laboratory Evaluation

C-reactive protein levels, platelets, neutrophils, and lymphocytes on the same day as the colonoscopy were recorded in all participants. SII measured for all patients. SII and CRP values were compared between UC patients with active disease or remission. The correlations between CRP, SII, and Mayo endoscopic subscore were analyzed. In addition, ROC curve analysis for SII was performed to determine the cut-off value, sensitivity, and specificity in determining ulcerative colitis activity.

### 2.5. Exclusion Criteria

Age under 18 years, history of intestinal surgery, presence of active infection, coexistent hepatic and/or renal failure, presence of chronic disorders, pregnancy, lactation, proctitis, patients with hematological disease, malignancy, patients using biological agents, and patients without laboratory values on the same day as colonoscopy were excluded from the study.

### 2.6. Statistical Analysis

Statistical analysis of the study was done by using SPSS 25.0 (IBM Statistical Package for Social Sciences software version 25). Continuous variables were expressed as a mean ± standard deviation and categorical variables as a percentage. Chi-square test was used to compare categorical values, and the Mann-Whitney *U* test was used to compare continuous variables between groups. Receiver-operating characteristic (ROC) analysis was performed to calculate the cut-off values. Correlations between SII, CRP, and Mayo endoscopic subscore were determined using Spearman's rho test. A *p* value of less than 0.05 was considered as statistically significant.

### 2.7. Ethical Considerations

The study protocol was approved by the local ethics committee (approval no.: 2022-0032).

## 3. Results

A total of 81 ulcerative colitis patients, 42 (51.2%) female and 39 (48.8%) male, were included in the study. The mean age was 44.0 ± 15.20 years in active disease and 46.9 ± 13.5 years in remission. While 47 (58%) of the patients were in the active period, 34 (42%) were in remission. There was no difference between active and remission patients in terms of gender, age, site of involvement, and the treatment they received. While the Mayo subscore was 0 in all patients in remission, it was 1 in 17, 2 in 18, and 3 in 12 active patients. The comparison between patients with active ulcerative colitis and patients in remission is summarized in Table 1.

### 3.1. Evaluation of Laboratory Results

The mean CRP levels were 22 mg/dl in ulcerative colitis patients with active disease and 1.1 mg/dl in remission patients (*p* < 0.001).

SII levels were significantly different between active and remission groups (1497 ± 1300 and 495 ± 224, *p* < 0.001, respectively).

In the correlation analysis, a significant correlation was found between SII, CRP, and Mayo subscores. Comparison of laboratory results and correlation analysis between groups is summarized in Table 2.

**Figure 1:**
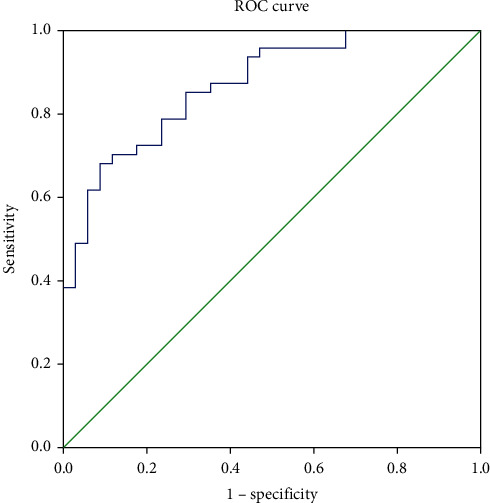
ROC curve analysis of SII values in the patients with active ulcerative colitis.

In ROC curve analysis, SII was found to be significantly effective in determining activity in ulcerative colitis patients. For 0.860 area under curve, the sensitivity was 68.1% and the specificity was 91.2% at a cut-off value of 781.5 (Figure 1).

## 4. Discussion

The role of neutrophils in the pathogenesis of ulcerative colitis is well known. With the massive infiltration and activation of neutrophils into the inflamed area, proteinase and matrix metalloproteinases are excessively released and oxygen radicals increase. This causes crypt damage. As neutrophils increase in the lamina propria, the crypt epithelium is more commonly damaged and causes ulceration in the mucosa [15, 16]. On the other hand, there is dysregulation in neutrophil apoptosis in ulcerative colitis. Antiapoptotic cytokines such as granulocyte-macrophage colony-stimulating factor are shown as the cause of this situation. Thus, neutrophils stay in the mucosal inflammation area for a long time and accumulate, causing a delay in the clearance of inflammation [17]. Therefore, the disease activity in ulcerative colitis is parallel to the increase of neutrophils.

It is also known that inflammation in ulcerative colitis is associated with platelets. In active ulcerative colitis, platelet counts increase and there are morphological changes such as increase in size, loss of discoid shape, increase in granular contents, and increase in density. Overproduction and release of many factors such as fibrinogen, P-selectin, von Willebrand factor, and fibrinolitic inhibitors occur from the granular content. There is an increase in the number of receptors associated with cytokines and complement components in the platelet membrane [18]. The result is an increase in the number and activity of platelets in ulcerative colitis. An increase in platelet count is accepted as a biomarker of activation in inflammatory bowel diseases [19, 20].

It has been shown in many studies that SII value is an indicator of poor prognosis in malignancies where the inflammatory reaction is active [21]. Again, it has been demonstrated that SII is a significant activation indicator in active rheumatic diseases in which there is a strong inflammatory response [22]. In this case, as the severity of inflammation increases, the evaluation of SII as an activation marker comes to the fore in ulcerative colitis with increased activity. SII obtained using platelet, neutrophil, and lymphocyte counts can be a strong indicator of inflammatory status in ulcerative colitis patients.

In our study, we compared SII values between patients with active ulcerative colitis and patients in remission, with the hypothesis that the SII value will increase as the inflammation activity increases. We found the mean SII values to be significantly higher in active patients than in patients in remission (1497 ± 1300 and 495 ± 224, *p* < 0.001, respectively). As expected, CRP and Mayo score were significantly higher in active disease (*p* < 0.001). We found a significant positive correlation between SII, CRP, and Mayo scores. In our study, the sensitivity and specificity of SII were 68.1% and 91.2% at the cut-off value of 781.5.

There are two studies in the literature that evaluated SII in ulcerative colitis patients. In the study published by Xie et al. in 2021, SII was evaluated in ulcerative colitis patients and in the control group, and it was found to be significantly associated with disease activity [23]. Similar to our study, a significant positive correlation was found between SII, Mayo score, and CRP in the correlation analysis. In the study published by Zhang et al. in 2021, SII values in ulcerative colitis patients were found to be significantly higher than the control group and correlated with the Mayo score [24]. On the basis of these findings, we think that SII value is higher in active disease due to the increase in platelet and neutrophil counts with activity in accordance with the pathogenesis of ulcerative colitis. In the light of the results obtained from previous studies and the current study, we think that the use of SII as a noninvasive and radiation-free activity marker in the follow-up of inflammatory bowel disease will be beneficial.

There are some limitations in our study. First of all, the study was done retrospectively. A prospective study with larger patient groups would be more powerful. In addition, histological severity was not evaluated in patients, and therefore, histological severity was not compared with SII values. Because of their small number, patients with proctitis were not included in the study. There was no control group in our study, and the comparison was made between active and remission patients.

In conclusion, SII is an indicator of a strong inflammatory response. Patients with active ulcerative colitis, who have a stronger inflammatory response, have significantly higher SII values than those in remission. It shows promise for use as a noninvasive marker of active ulcerative colitis. These results need to be supported by prospective studies in large patient groups.

## Figures and Tables

**Table 1:** Comparison of demographic, colonoscopic, and laboratory findings between the patients with active ulcerative colitis and ulcerative colitis in remission.

	Patients with active UC *n* = 47	Patients with UC in remission *n* = 34	*p*
Age (year)	44.0 ± 15.20	46.9 ± 13.5	0.379
Sex (F/M)	25/22	17/17	0.477
Site of involvement			0.85
Left-sided	22	22	
Extensive	25	12	
Treatment			
None	3	0	0.26
5-ASA	30	23	0.815
Azatiopurine	10	11	0.309
Steroid	4	0	0.135
Mayo subscore			<0.001
0	0	34	
1	17	0	
2	18	0	
3	12	0	

UC: ulcerative colitis; ASA: acetylsalicylic acid.

**Table 2:** Correlation analysis and comparison of laboratory findings between the patients with active ulcerative colitis and ulcerative colitis in remission.

	Patients with active UC *n* = 47	Patients with UC in remission *n* = 34	*p*
SII	1497 ± 1300	495 ± 224	<0.001
CRP (mg/dl)	22 (1.7-161)	1.1 (0-5.1)	<0.001
Correlation analysis			
SII-CRP			<0.001
SII-Mayo subscore			0.004

UC: ulcerative colitis; SII: systemic immune-inflammatory index; CRP: C-reactive protein.

## Data Availability

The data used to support the findings of this study are available from the corresponding author upon request.
